# MecVax supplemented with CFA MEFA-II induces functional antibodies against 12 adhesins (CFA/I, CS1–CS7, CS12, CS14, CS17, and CS21) and 2 toxins (STa, LT) of enterotoxigenic *Escherichia coli* (ETEC)

**DOI:** 10.1128/spectrum.04153-23

**Published:** 2024-02-16

**Authors:** Chongyang Zhang, Siqi Li, Ipshita Upadhyay, Kathyrn L. Lauder, David A. Sack, Weiping Zhang

**Affiliations:** 1 Department of Pathobiology, University of Illinois at Urbana-Champaign, Urbana, Illinois, USA; 2 Department of International Health, Johns Hopkins University Bloomberg School of Public Health, Baltimore, Maryland, USA; Tainan Hospital, Ministry of Health and Welfare, Tainan City, Taiwan

**Keywords:** ETEC (enterotoxigenic *E. coli*), diarrhea, vaccine, MecVax, CFA MEFA-II

## Abstract

Enterotoxigenic *Escherichia coli* (ETEC) strains that produce various adhesins and one or two enterotoxins are the leading causes of children’s diarrhea and travelers’ diarrhea. MecVax, a multivalent ETEC vaccine candidate, consists of two proteins, an adhesin multiepitope fusion antigen (MEFA) that stimulates antibodies to the seven most important ETEC adhesins (CFA/I and CS1–CS6) and a toxoid fusion antigen which stimulates antibodies against ETEC enterotoxins (heat-labile toxin and heat-stable toxin). CFA MEFA-II, another polyvalent MEFA protein, has been demonstrated to stimulate antibodies to another five important ETEC adhesins (CS7, CS12, CS14, CS17, and CS21). We hypothesize that MecVax coverage and efficacy can be expanded if MecVax could stimulate antibodies to all 12 adhesins. In this study, we supplemented MecVax with CFA MEFA-II, examined broad immunity to the 12 targeted ETEC adhesins and 2 ETEC toxins (STa, LT) in mice, and assessed mouse antibody functions for inhibiting the adherence of the 12 adhesins and neutralizing the enterotoxicity of 2 toxins, thus assessing the potential application of a broadly protective pan-ETEC vaccine. Mice intramuscularly immunized with MecVax and CFA MEFA-II developed robust antibody responses to the 12 ETEC adhesins and 2 toxins; furthermore, mouse serum antibodies showed functional activities against the adherence from each of the targeted adhesins and the enterotoxicity of either toxin. Data also indicated that CFA MEFA-II was antigenically compatible with MecVax. These results demonstrated that the inclusion of CFA MEFA-II further expands MecVax broad immunogenicity and protection coverage, suggesting the feasibility of developing a vaccine against all important diarrheal ETEC strains.

**IMPORTANCE:**

There are no vaccines licensed for Enterotoxigenic *Escherichia coli* (ETEC), a leading cause of children’s diarrhea and the most common cause of travelers’ diarrhea. Since ETEC strains produce over 25 adhesins and 2 distinctive enterotoxins, heterogeneity is a key obstacle to vaccine development. MecVax, a multivalent ETEC vaccine candidate, induces protective antibodies against the seven most important adhesins (CFA/I and CS1–CS6) associated with two-thirds of ETEC clinical cases. However, ETEC prevalence shifts chronically and geographically, and other adhesins are also associated with clinical cases. MecVax would become a pan-ETEC vaccine if it also protects against the remaining important adhesins. This study demonstrated that MecVax supplemented with adhesin protein CFA MEFA-II induces functional antibodies against 12 important ETEC adhesins (CFA/I, CS1–CS7, CS12, CS14, CS17, and CS21), enabling the development of a more broadly protective ETEC vaccine and further validating the application of the MEFA vaccinology platform for multivalent vaccine development.

## INTRODUCTION

Enterotoxigenic *Escherichia coli* (ETEC) strains that produce heterogeneous adhesins [colonization factor antigen (CFA) or coli surface antigen (CS)] and two types of enterotoxins [heat-labile toxin (LT) and heat-stable toxin (STa)], are one of the top four causes of diarrhea in children living in developing countries (children’s diarrhea) and the most common course of diarrhea in international travelers (travelers’ diarrhea) (1–4). Currently, there is no vaccine licensed against ETEC-associated children’s diarrhea or travelers’ diarrhea. An effective ETEC vaccine can potentially save about a hundred thousand lives and prevent over 200 million clinical diarrhea cases annually (5–7). Additionally, a protective ETEC vaccine would reduce the use of antibiotics to treat patients with severe infections from ETEC strains which continuously acquire antibiotic resistance, thus helping to combat the emergence of antimicrobial resistance and superbugs (8, 9).

One key challenge in developing effective vaccines for ETEC is the heterogeneity among ETEC strains (pathovars). ETEC strains possess different profiles of virulence factors, with various combinations of adhesins and toxins. There are over 25 immunologically heterogeneous adhesins, including CFA and CS, produced by ETEC strains to attach bacteria to host receptors and colonize small intestines. These ETEC strains produce one or two types of enterotoxins, LT, and STa and deliver toxins to intestinal epithelial cells to elevate intracellular cyclic adenosine monophosphate (cAMP) or guanosine monophosphate (cGMP) levels and cause fluid hyper-secretion into the gut lumen. Epidemiological and systematic prevalence studies revealed that the ETEC strains expressing seven adhesins, CFA/I and CS1–CS6 (and STa and/or LT toxin), are estimated to cause two-thirds of the ETEC-associated clinical cases as well as the moderate-to-severe cases (10–12). However, ETEC prevalence can shift chronically and geographically (10, 13). ETEC strains producing other adhesins including CS7, CS12, CS14, CS17, and CS21 (and one or both ETEC toxins) also play a significant role in causing clinical diarrhea and moderate-to-severe diarrheal cases (10, 11, 14).

There are a few ETEC vaccine candidates currently under preclinical or clinical investigation, but past ETEC vaccine research and development efforts mostly target a few of the seven most prevalent adhesins (CFA/I and CS1–CS6) in addition to toxin LT but barely STa (15). Among them, ETVAX, a killed whole-cell ETEC vaccine candidate, composed of four inactivated strains to express four adhesins (CFA/I, CS3, CS5, and CS6) and supplemented with a recombinant CT_B_/LT_B_ chimeric protein (16), was demonstrated to be tolerated and induce moderate cross-reactive immunity in adults and children (17–19). However, ETVAX does not carry antigens to protect against STa, the ETEC toxin that plays a more important role in causing children’s diarrhea and travelers’ diarrhea (20, 21). In contrast to the conventional cellular and acellular vaccine approaches, a novel epitope- and structure-based vaccinology platform named multiepitope-fusion-antigen (MEFA) was developed recently and used to construct a multivalent ETEC vaccine candidate MecVax to target all of the seven most important ETEC adhesins and both ETEC toxins (22–25). MecVax is composed of two polyvalent protein immunogens that present heterogeneous functional epitopes (from seven ETEC adhesins or two ETEC toxins) on a backbone and mimic epitope native antigenicity. We have demonstrated that MecVax not only induces functional antibodies against the seven targeted adhesins (CFA/I and CS1–CS6) and two toxins (STa, LT) but also protects against ETEC intestinal colonization and clinical diarrhea in animal models (23, 24, 26, 27). More recently, by using the same MEFA platform, we generated another polyvalent MEFA protein (termed as CFA MEFA-II) to target five remaining important ETEC adhesins (CS7, CS12, CS14, CS17, and CS21) and showed that this CFA MEFA-II protein elicits functional antibodies against the five targeted ETEC adhesins (28, 29).

An ideal ETEC vaccine would cover the important ETEC adhesins and both ETEC toxins and protect against all cases of ETEC-associated children’s diarrhea and travelers’ diarrhea. MecVax can become a pan-ETEC vaccine if it can further expand coverage and induce functional antibodies against the 12 important ETEC adhesins (CFA/I, CS1–CS6, CS7, CS12, CS14, CS17, and CS21) and two ETEC toxins (STa, LT). In this study, we combined three polyvalent proteins, by supplementing MecVax with adhesin MEFA-II, intramuscularly immunized mice, examined mouse antigen-specific antibody responses, and measured mouse antibody functional activities against ETEC bacterial adherence and toxin enterotoxicity. Results showed that the immunized mice developed robust antibody responses to all target antigens, 12 ETEC adhesins and 2 ETEC toxins. Moreover, the induced antibodies were functional, as shown by the inhibition of adherence of bacteria that express any of the target adhesins and neutralization of both toxins. While the current study is limited to immunogenicity and antibody functional activity assessment and future studies of preclinical and clinical efficacy evaluation are needed, data from this study suggest the potential of MecVax combined with CFA MEFA-II for developing a more broadly protective vaccine against ETEC-associated diarrhea.

## RESULTS

### MecVax supplemented with CFA MEFA-II (MecVax + CFA MEFA-II) induced robust antibody responses to 12 ETEC adhesins and both ETEC toxins in mice

**Fig 1:**
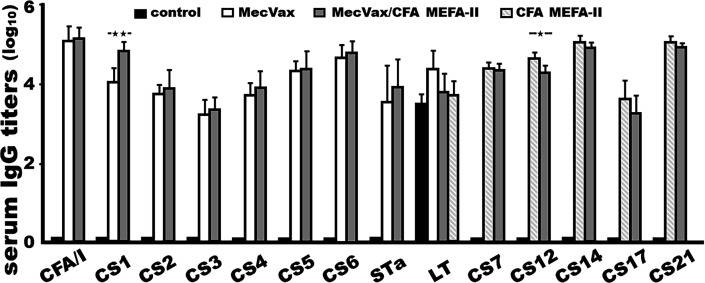
Mouse serum antigen-specific IgG titers (log_10_) from the group intramuscularly immunized with MecVax (white boxes), “MecVax + CFA MEFA-II” (MecVax supplemented with CFA MEFA-II; gray boxes), CFA MEFA-II (white boxes with diagonal lines), or phosphate buffer saline (PBS) (dark boxes); dmLT adjuvant was included in four groups. ELISAs with 2HB plates coated with 100 ng recombinant protein CfaB, CooA, CotA, CstH, CsaB, CsfA, CssA, CsvA, CswA, CsuA, CsbA, LngA, or cholera toxin (CT, Sigma) or CoStar plates with 10 ng STa-ovalbumin conjugates were used to titrate anti-CFA/I, CS1, CS2, CS3, CS4, CS5, CS6, CS7, CS12, CS14, CS17, CS21, LT, or STa IgG antibodies in twofold serum dilutions (1:400 to 1:102,400) from each mouse in a group. Antibody titers (in log_10_) were presented in means and standard deviations. * and ** indicate a *P*-value less than 0.05 and 0.001, respectively.

Mice intramuscularly (IM) immunized with MecVax combined with CFA MEFA-II (“MecVax + CFA MEFA-II”) developed robust antibody responses to all targeted antigens, 12 ETEC adhesins and 2 ETEC toxins (Fig. 1). Anti-CFA/I, -CS1, -CS2, -CS3, -CS4, -CS5, -CS6, -CS7, -CS12, -CS14, -CS17, -CS21, -STa, and anti-LT IgG titers (log_10_) were detected at 5.2 ± 0.26, 4.9 ± 0.18, 3.9 ± 0.44, 3.4 ± 0.28, 4.0 ± 0.41, 4.4 ± 0.48, 4.8 ± 0.25, 4.4 ± 0.15, 4.3 ± 0.14, 5.0 ± 0.10, 3.3 ± 0.43, 5.0 ± 0.06, 3.9 ± 0.68, and 3.7 ± 0.45 (log_10_), respectively, from the serum samples of the mice IM immunized with “MecVax + CFA MEFA-II.”

No antigen-specific antibodies were detected from the control mice (immunized with PBS and dmLT adjuvant) except for anti-LT antibodies (3.4 ± 0.34; log_10_). No antigen-specific antibody responses were detected from any mouse serum samples (including sera of the control group) collected before the primary immunization.

### Mouse antibodies derived from the co-administration of MecVax and CFA MEFA-II (“MecVax + CFA MEFA-II”) inhibited adherence from 12 ETEC adhesins (CFA/I, CS1–CS7, CS12, CS14, CS17, and CS21)

**Fig 2:**
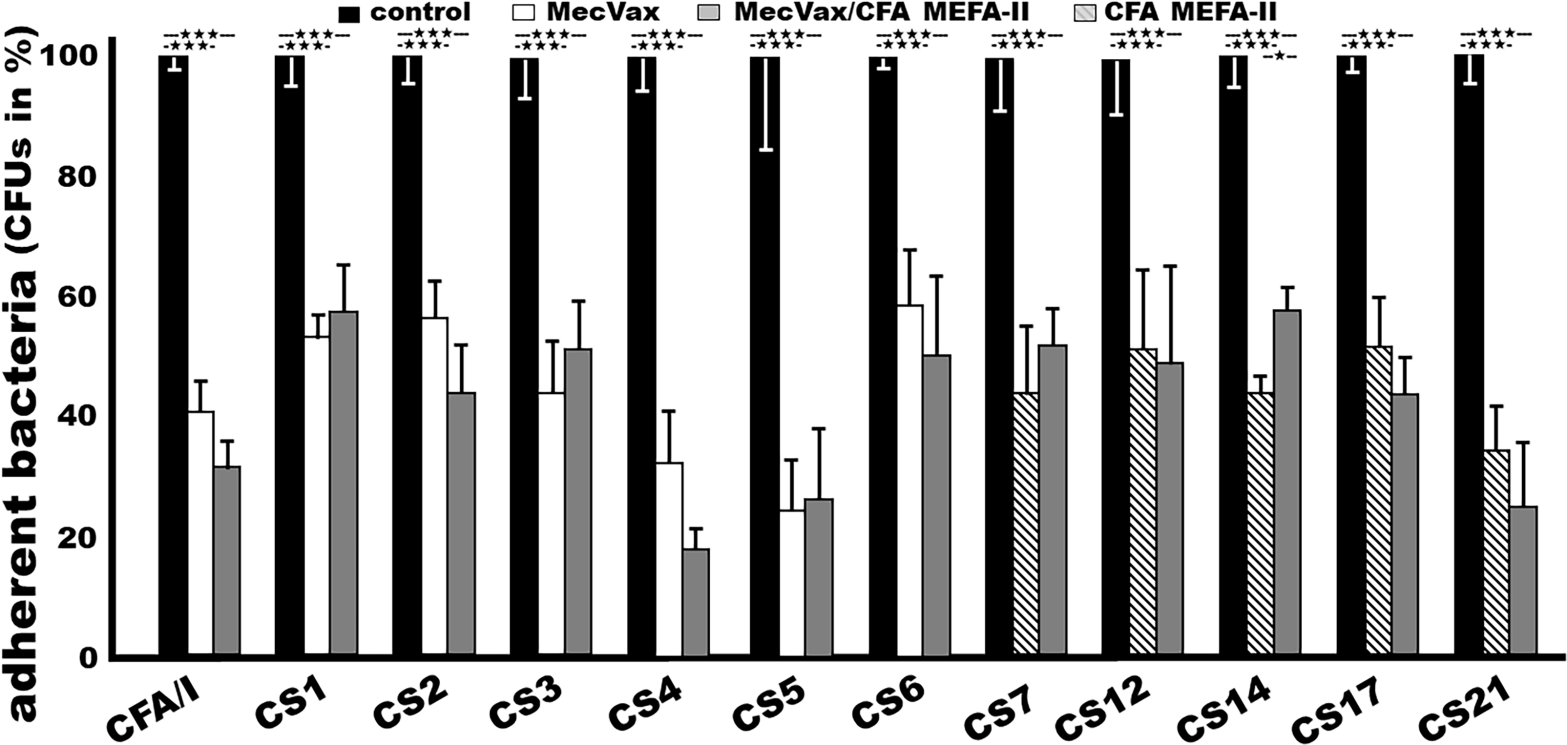
Results of antibody inhibition assay to show mouse serum antibodies inhibited adherence of *E. coli* (CS1, CS2) or ETEC strains expressing the adhesins targeted by MecVax (CFA/I, CS1–CS6) and CFA MEFA-II (CS7, CS12, CS14, CS17, and CS21) to Caco-2 cells. Adherent (to Caco-2 cells) bacteria expressing CFA/I, CS1, CS2, CS3, CS4/CS6, CS5/CS6, CS6, CS7, CS12/CS20, CS14, CS17, or CS21 adhesin, after treated with mouse sera from the group injected with PBS (dark boxed), MecVax (white boxes), “MecVax + CFA MEFA-II” (gray boxes), or CFA MEFA-II (white boxes with diagonal lines), were counted (CFUs; in means and standard deviations) and converted to percentages by referring CFUs from cells treated with control mouse sera as 100%. *** indicates a *P* value less than 0.0001.

Adherence to Caco-2 cells by ETEC or recombinant (CS1, CS2) bacteria that express the targeted adhesins was significantly inhibited after incubation with the serum samples of the mice IM immunized with “MecVax + CFA MEFA-II” (Fig. 2), indicating the induced adhesin-specific antibodies are functional. ETEC H10407 (CFA/I), THK38/pEU405 (CS1), DH5a/pEU588 (CS2), E116 (CS3), E106 (CS4/CS6), UM75688 (CS5/CS6), 2423 ETP98066 (CS6), JF2327 (CS7), JF3276 (CS12/CS20), JF2125 (CS14), JF2350 (CS17), and JF2101 (CS21) bacterial adherence (measured in CFUs) was reduced by 69 ± 4.3, 43 ± 7.6, 56 ± 7.8, 49 ± 7.8, 82 ± 2.8, 74 ± 11.3, 50 ± 12.5, 48 ± 6.2, 51 ± 16.2, 42 ± 3.5, 56 ± 5.8, and 74 ± 10.7 (%), respectively, after bacteria were incubated with the sera from the mice immunized with “MecVax + CFA MEFA-II,” compared with the adherent bacteria after incubation with the sera from the control mice.

### Mouse antibodies derived from ‘MecVax + CFA MEFA-II’ neutralized the enterotoxicity of both ETEC toxins (STa, CT)

**Fig 3:**
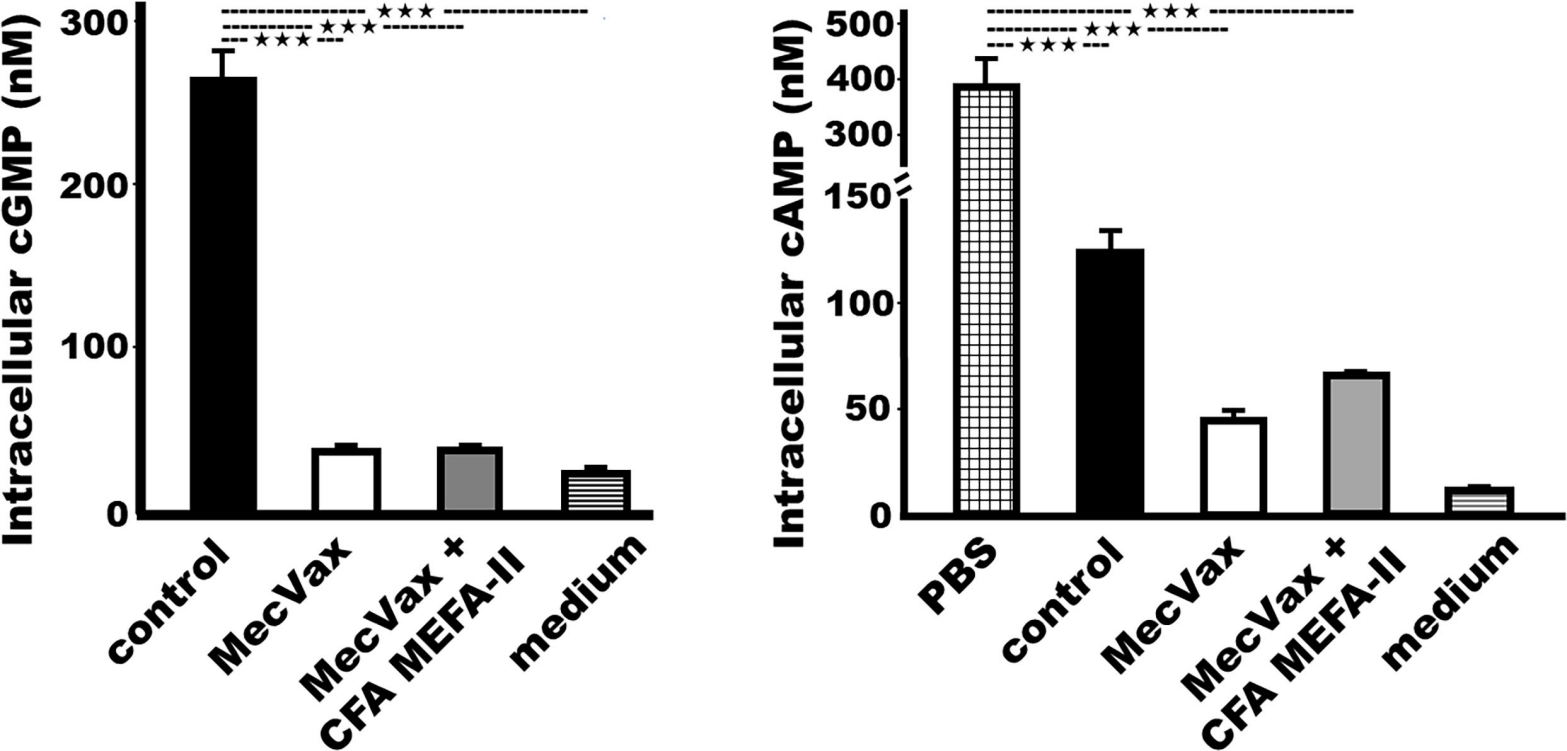
Results of antibody neutralization activities against STa or CT enterotoxicity, by using T-84 cells and a cyclic GMP or AMP EIA kit. Intracellular cGMP (left) or cAMP (right) concentrations (nM; in means and standard deviations) in T-84 cells after incubation with toxin STa (2 ng) or CT (30 ng) which were pre-treated with mouse sera pooled from the group IM immunized with PBS control (black box), MecVax (white box), or “MecVax + CFA MEFA-II” (gray box). Intracellular cGMP or cAMP levels of T-84 cells incubated with culture medium (no toxin and no sera) were used as the baseline (a box with horizontal lines). *** indicates a *P*-value less than 0.0001.

Sera from the mice IM immunized with “MecVax + CFA MEFA-II” neutralized STa and CT enterotoxicity, shown by the prevention of STa or CT enterotoxicity from elevating intracellular cGMP or cAMP in T-84 cells (Fig. 3). T-84 cell intracellular cGMP levels were 39 ± 3.4 (nM) after cells were incubated with STa toxin (2 ng) pre-mixed with the sera from the mice immunized with “MecVax + CFA MEFA-II.” In contrast, significantly greater cGMP levels (266 ± 17.1 nM; *P* < 0.0001) were detected in the T-84 cells that were incubated with STa pre-treated with the control mouse sera. The baseline intracellular cGMP levels (T-84 cells in tissue culture medium) were 24 ± 3.7 nM.

The intracellular cAMP levels in the cells incubated with CT (cholera toxin, a homolog of LT, 30 ng) pre-mixed with the sera from the mice immunized with “MecVax + CFA MEFA-II” were 76 ± 2.0 nM. These levels were significantly lower than the cAMP in the cells treated with CT only (394 ± 50.1 nM; *P* < 0.0001). Because dmLT adjuvant was administered to all the mice including the control group and dmLT induces neutralizing anti-LT antibodies, the cAMP concentrations in the cells treated with CT and the control mouse sera were 124 ± 10.3 nM, which were higher than the levels in the cells treated with sera of the mice immunized with “MecVax + CFA MEFA-II” though the increasing was not significant. The baseline cAMP levels in T-84 cells (in tissue culture medium, no serum or toxin) were 12 ± 0.4 nM.

### MecVax and CFA MEFA-II were antigenically compatible for IM immunization in mice

Mice IM immunized with MecVax or “MecVax + CFA MEFA-II” developed similar levels of antibody responses to the antigens of MecVax, seven adhesins and two toxins (Fig. 1). Anti-CFA/I, -CS1, -CS2, -CS3, -CS4, -CS5, -CS6, -STa, and anti-LT IgG titers were detected at 5.1 ± 0.37, 4.1 ± 0.32, 3.8 ± 0.22, 3.3 ± 0.30, 3.8 ± 0.28, 4.3 ± 0.22, 4.7 ± 0.32, 3.5 ± 0.88, and 4.3 ± 0.43 (log_10_), respectively, from the sera of the mice immunized with MecVax alone. These antigen-specific IgG titers were not significantly different from those in the group immunized with “MecVax + CFA MEFA-II,” except for anti-CS1 titers of which the mice immunized with MecVax showed a lower titer (4.1 ± 0.32; *P* < 0.001), compared with the group immunized with “MecVax + CFA MEFA-II” (4.9 ± 0.18).

Similarly, mice in the group immunized with “MecVax + CFA MEFA-II” and the group immunized with CFA MEFA-II developed similar IgG titers to CS7, CS12, CS14, CS17, or CS21, the adhesins targeted by CFA MEFA-II (Fig. 1). Anti-CS7, -CS12, -CS14, -CS17, and anti-CS21 IgG titers in the sera of the mice IM immunized with CFA MEFA-II were 4.4 ± 0.12, 4.7 ± 0.15, 5.1 ± 0.16, 3.6 ± 0.46, and 5.1 ± 0.12 (log_10_), respectively. The anti-CS7, -CS14, -CS17, and anti-CS21 IgG titers were not significantly different than the titers in the mice immunized with “MecVax + CFA MEFA-II” (4.4 ± 0.15, 5.0 ± 0.10; 3.3 ± 0.43, and 5.0 ± 0.06). Only anti-CS12 titers in the group immunized with CFA MEFA-II were higher (4.7 ± 0.15; *P* < 0.05) than the group immunized with “MecVax + CFA MEFA-II” (4.3 ± 0.14).

### Mouse serum antibodies derived from “MecVax + CFA MEFA-II” or from MecVax equally inhibited the adherence of CFA/I and CS–CS6 adhesins

Serum antibodies derived from the group immunized with “MecVax + CFA MEFA-II” or the group immunized with MecVax displayed the same levels of adherence inhibition activities against the seven targeted ETEC adhesins (CFA/I and CS1–CS6) (Fig. 2). After incubation with sera from the mice immunized with MecVax, ETEC or *E. coli* bacteria H10407 (CFA/I), THK/pEU405 (CS1), DH5a/pEU588 (CS2), E116 (CS3), E106 (CS4/CS6), UM75688 (CS5/CS6), and 2423 ETP98066 (CS6) adherence was reduced by 60 ± 4.9, 47 ± 3.8, 44 ± 6.0, 56 ± 8.7, 68 ± 8.4, 76 ± 8.1, and 41 ± 9.2 (% of CFUs), respectively. The adherence reduction to each of these seven ETEC strains was not significantly different when they were incubated with mouse sera from the group immunized with MecVax or sera from the group immunized with “MecVax + CFA MEFA-II” (69 ± 4.3, 43 ± 7.6, 56 ± 7.8, 49 ± 7.8, 82 ± 2.8, 74 ± 11.3, and 50 ± 12.5).

### Mouse serum antibodies derived from “MecVax + CFA MEFA-II” or CFA MEFA-II equally inhibited the adherence of CS7, CS12, CS14, CS17, and CS21 adhesins

Serum antibodies derived from the group immunized with “MecVax + CFA MEFA-II” or the group immunized with CFA MEFA-II showed the same levels of adherence inhibition activities against the five targeted ETEC adhesins (CS7, CS12, CS14, CS17, and CS21) (Fig. 2). After incubation with the sera from the mice immunized with CFA MEFA-II alone, JF2327 (CS7), JF3276 (CS12/CS20), JF2125 (CS14), JF2350 (CS17), or JF2101 (CS21) adherence to Caco-2 cells was reduced by 56 ± 10.8, 48 ± 12.6, 54 ± 3.1, 48 ± 7.9, and 65 ± 7.0 (CFUs, in %), respectively. These reductions in adherence to CS7, CS12, CS14, CS17, or CS21 by the serum antibodies from the mice immunized with CFA MEFA-II were not significantly different from the reduction by the mouse sera from the group immunized with “MecVax + CFA MEFA-II” (48 ± 6.2, 51 ± 16.2, 42 ± 3.5, 56 ± 5.8, and 74 ± 10.7).

### Mouse serum antibodies derived from “MecVax + CFA MEFA-II” or MecVax equally neutralized STa or CT enterotoxicity

The sera from the mice IM immunized with MecVax or “MecVax + CFA MEFA-II” showed similar levels of neutralizing activities against STa or CT enterotoxicity (Fig. 3), indicating that toxin-specific antibody neutralization activities from MecVax (which carries toxin antigens) were not compromised after being combined with CFA MEFA-II protein antigen. The cGMP concentrations in T-84 cells incubated with STa and the sera from the group immunized with MecVax were 38 ± 2.6 nM, which is nearly identical to the cGMP levels in the cells treated with the toxin and the sera of the mice immunized with “MecVax + CFA MEFA-II” (39 ± 3.4 nM). Similarly, the cAMP in the T-84 cells treated with CT and the sera from the group immunized with MecVax (45 ± 4.4 nM) did not differ significantly from the cAMP levels in the cells treated with CT and sera of the group immunized with “MecVax + CFA MEFA-II” (76 ± 2.0 nM; *P* = 0.54).

## DISCUSSION

Data from this study indicated that mice intramuscularly immunized with MecVax supplemented with polyvalent antigen CFA MEFA-II (“MecVax + CFA-MEFA-II”) developed functional antibodies unprecedentedly to 12 ETEC adhesins (CFA/I, CS1–CS7, CS12, CS14, CS17, and CS21) and 2 toxins (STa, LT). These 12 adhesins (together with one or both enterotoxins) are expressed by ETEC strains causing more than 86% of ETEC clinical diarrheal cases and moderate-to-severe cases. A vaccine that induces protective immunity against adherence to these 12 ETEC adhesins can potentially protect against ETEC-associated moderate-to-severe cases and a vast majority of clinical diarrheal cases.

Results from this study indicate that the third protein antigen CFA MEFA-II is antigenically compatible with the two proteins of MecVax. Mice IM immunized with “MecVax + CFA MEFA-II” developed similar levels of antibody responses to eight of the nine targeted antigens (CFA-I, CS2–CS6, STa, and LT) as the mice IM immunized with MecVax. Similarly, mice immunized with “MecVax and CFA MEFA-II” and the mice IM immunized with CFA MEFA-II developed similar levels of antibody responses to four of the five targeted antigens (CS7, CS14, CS17, and CS21). More importantly, antibodies elicited by “MecVax + CFA MEFA-II” and the antibodies derived from MecVax had the same level of protection against adherence by ETEC strains expressing any of the seven target adhesins. Similarly, antibodies induced by “MecVax and CFA MEFA-II” and antibodies elicited by CFA MEFA-II equally inhibited the adherence of the five targeted adhesins. Additionally, the antitoxin antibodies derived from MecVax, alone or along with CFA MEFA-II, had the same level of neutralization activities against STa toxin, indicated by nearly identical levels of cGMP in T-84 cells after treatment with STa toxin and the sera from either immunization group (“MecVax + CFA MEFA-II” versus MecVax). The anti-LT antibody response and anti-LT (anti-CT) antibody neutralization activity were detected at the same levels from the group immunized with “MecVax and CFA MEFA-II” or the group immunized with MecVax alone, though the similarities were not conclusive in the current study because of the additive effect from adjuvant dmLT that also induces functional anti-LT antibodies. However, MecVax neutralization against CT enterotoxicity and protection from LT-mediated diarrhea were demonstrated previously (23–25). While protective anti-LT antibodies from dmLT adjuvant is an additive to MecVax for protection against LT-producing ETEC infection, future studies using a non-LT adjuvant will allow us to conclusively evaluate if anti-LT antibody response and neutralization activity against LT (CT) are compromised after MecVax is co-administered with CFA MEFA-II or another polyvalent protein antigen.

MecVax is a protein-based multivalent ETEC vaccine candidate that has been demonstrated to induce functional antibodies against the seven most important ETEC adhesins (CFA/I and CS1–CS6) and two ETEC toxins (STa, LT) (24, 25, 30). Moreover, MecVax is shown to protect against ETEC toxin-mediated clinical diarrhea in a pig challenge model and prevent over 99% of ETEC bacterial colonization in small intestines by ETEC strains expressing any of the seven targeted adhesins in a rabbit model (26, 27). The adhesin-specific antibodies elicited by MecVax can potentially prevent over two-thirds of ETEC-associated clinical diarrheal cases since ETEC strains producing CFA/I and CS1–CS6 adhesins are estimated to be associated with about 65% of clinical cases (10, 14). When supplemented with CFA MEFA-II which targets another five important ETEC adhesins (CS7, CS12, CS14, CS17, and CS21), MecVax expands its coverage to 12 ETEC adhesins, including all important ETEC adhesins associated with moderate-to-severe diarrheal cases. The functional antibodies against 12 adhesins can potentially improve MecVax efficacy from 65% to over 86% since CFA/I, CS1–CS7, CS12, CS14, CS17, and CS21 adhesins are expressed by 86.1% of STa-positive ETEC strains, 80.6% of STa- and/or LT-positive ETEC strains, and 47.3% of LT-positive ETEC strains (14). In addition, the toxin-specific antibodies elicited by MecVax neutralize STa and LT, the two enterotoxins, alone or together, are produced by all ETEC strains. This antitoxin immunity from the improved MecVax can provide supplemental (to the anti-adhesin immunity) protection against ETEC strains producing any of the 12 targeted adhesins and also independent protection against all the other ETEC strains that do not express the 12 targeted adhesins, further expanding vaccine protection against ETEC diarrhea.

It needs to be pointed out that the current study examined only the vaccine’s broad immunogenicity and antibody *in vitro* functional activities against ETEC adherence and toxin excitotoxicity. Future studies with a rabbit colonization model and a pig passive protection model will allow us to evaluate the efficacy of the new MecVax preclinically. Surely, the controlled human infection model and clinical trials will eventually validate the broad immunogenicity and cross-protection of this protein-based injectable ETEC vaccine candidate.

The demonstration of no or negligent effects at antigen-specific immunogenicity and more importantly protective functions after multiple polyvalent protein antigens were combined and co-administered from this study not only signifies the feasibility of constructing a more effective ETEC vaccine but also paves the way toward future preparation of combination vaccines against different diseases. Combination vaccines can simplify the logistics of vaccine manufacture, storage, and administration, reducing vaccine costs and clinical expenses. Combination vaccines will become even more desirable as the expanded program on immunization especially for children is getting increasingly crowded because of the continuous introduction of new vaccines. We recently demonstrated that a polyvalent *Shigella* or cholera MEFA protein antigen is broadly immunogenic and cross-protective against heterogeneous serogroup or serotype strains (31, 32). The encouraging data from the current study certainly enhance confidence for future research and development of combination vaccines against ETEC and *Shigella* and/or cholera (or other enteric pathogens), particularly since these pathogens infect the same populations, children in the endemic regions or countries as well as international travelers to these areas.

## MATERIALS AND METHODS

### Bacterial strains

ETEC field isolates or recombinant (CS1, CS2 only) strains expressing any of the 12 targeted adhesins were used to examine antibody functional activities against ETEC bacterial adherence. As described previously (24, 27–29), H10407 (CFA/I, LT, STa; Johns Hopkins University—JHU), THK38/pEU405 (CS1; Emory University), DH5a/pEU588 (CS2; Emory), E116 (CS3, STa, and LT; University of Gothenburg—GU), E106 (CS4, CS6, STa, and LT; GU), UM75688 (CS5, CS6, STa, and LT; JHU), 2423 ETP98066 (CS6, STa, and LT; University of Washington at St. Louis – WashU), JF2327 (CS7 and LT; WashU), JF3276 (CS12, CS20, STa, and LT; WashU), JF2125 (CS14 and LT; WashU), JF2350 (CS17 and LT; WashU), and JF2101 (CS21 and STa; WashU) were used for antibody adherence inhibition assays in this study.

Recombinant strains 9471 and 9472 were used to produce recombinant proteins 3xSTa_N12S_-mnLT_R192G/L211A_ and CFA/I/II/IV MEFA, the MecVax’s toxoid fusion antigen to target both ETEC toxins (STa and LT) and adhesin MEFA antigen to cover the seven most important adhesins (CFA/I and CS1–CS6) (23, 24). Recombinant strain 9916 was used to express CFA MEFA-II protein which targets five ETEC adhesins (CS7, CS12, CS14, CS17, and CS21) (28).

### Antigens and adjuvant

Four treatments including three immunization groups with different antigens and one control group were included in this study: “MecVax + CFA MEFA-II,” MecVax, CFA MEFA-II, or PBS. MecVax was composed of two recombinant proteins, toxoid fusion 3xSTa_N12S_-mnLT_R192G/L211A_ and adhesin antigen CFA/I/II/IV MEFA, 25 µg (in 10 µL) each. “MecVax + CFA MEFA-II” consisted of three proteins, 3xSTa_N12S_-mnLT_R192G/L211A_, CFA/I/II/IV MEFA, and CFA MEFA-II, 25 µg (in 10 µL) for each protein. The third treatment was of CFA MEFA-II only, 25 µg in 20 µL. The control group was composed of 25 µL PBS.

Holotoxin-structured double mutant LT (LT_R192G/L211A_; dmLT) (33), which differs from the mnLT_R192G/L211A_ component in the MecVax toxoid fusion antigen as this monomeric LT (mnLT) carries one A subunit mutant and one B subunit as a single peptide, 0.1 µg in 1 µL, was used as the adjuvant for four treatment groups in this study.

### Mouse intramuscular immunization

Eight-week-old BALB/c female mice (Charles River Laboratories, Wilmington, MA, USA), eight per group, were included in intramuscular immunization. The injection site at the quadriceps femoris was shaved or clipped, wiped with 70% ethanol, and intramuscularly injected with antigen(s) or PBS premixed with dmLT adjuvant, in a total volume of 21 or 31 µL, with a 25- or 30-gauge needle. Two booster injections at the same dose of the primary were followed at an interval of 2 weeks on the alternative body side. All mice were observed daily for activities and abnormalities.

Blood samples (30–50 μL) were collected from each mouse before the primary and each booster from the lateral saphenous vein with an 18- or 22-gauge needle. Two weeks after the second booster, mice were anesthetized with CO_2_ and euthanized with cervical dislocation. Blood samples (0.5 mL) were collected with cardiac puncture by ventral approach. Mouse sera were collected and stored at −20°C until use.

Mouse immunization protocol (#23060) was reviewed and approved by the Institutional Animal Care and Use Committee of the University of Illinois at Urbana-Champaign; animal studies were supervised by an institutional attending veterinarian and staff.

### Mouse serum antigen-antibody titration

Serum samples from each mouse were titrated in ELISAs for IgG antibody responses to the antigens targeted, 12 ETEC adhesins and 2 toxins. As we described previously (24, 25, 27–29), 100 ng recombinant protein of CFA/I structural subunit CfaB, CS1 structural subunit CooA, CS2 structural subunit CotA, CS3 structural and adhesive subunit CstH, CS4 structural subunit CsaB, CS5 structural subunit CsfA, CS6 structural subunit CssA, CS7 structural subunit CsvA, CS12 structural subunit CswA, CS14 structural subunit CsuA, CS17 structural subunit CsbA, or CS21 structural subunit LngA, in 100 µL 50 mM bicarbonate/carbonate coating buffer, was coated to each well of a 2HB 96-well microtiter plate (Thermo Fisher Scientific, Rochester, NY, USA) to titrate anti-adhesin antibody responses. To titrate antibody responses to LT or STa, 100 ng CT (Sigma, St. Louis, MO, USA) or 10 ng STa-ovalbumin conjugates, in 100 µL bicarbonate/carbonate buffer, was coated to each well of a 2HB plate or a CoStar 96-well plate (Thermo Fisher Scientific), respectively. After overnight growth at 4°C, coated plates were washed with phosphate-buffered saline with Tween-20 (PBST) (0.05% Tween-20), blocked with 10% non-fat milk (in PBST) at 37°C for 1 h, and incubated with twofold serum dilutions (1:400 to 1:102,400) at 37°C for 1 h. Wells were washed with PBST and incubated with horseradish peroxidase (HRP)-conjugated goat-anti-mouse IgG (1:5,000, Bethyl Laboratories, Montgomery, TX, USA) at 37°C for 1 h. Wells were washed with PBST and PBS and then incubated with 3,3′,5,5′-tetramethylbenzidine microwell peroxidase substrate system 2C (Thermo Fisher Scientific). Optical density (OD)_650_ readings were measured after 25 min at room temperature and subtracted with background values. The highest dilution given an OD over 0.3 was converted to a titer, at a scale of log_10_.

### Mouse serum antibody adherence inhibition against 12 ETEC adhesins

Sera pooled from eight mice in each group were examined for antibody activities against adherence from the 12 adhesins in antibody adherence inhibition assays. Since individual mouse serum samples from the same group showed similar levels of antigen-specific antibody responses and also the need for a high volume of sera for *in vitro* assays against 12 adhesins and 2 toxins, only pooled samples were used for antibody functional assays in this study. As described previously (24, 25, 27–29), 15 µL heat-inactivated serum sample pooled from the group immunized with “MecVax + CFA MEFA-II,” MecVax, or PBS (the control group) was mixed with 3.5 × 10^6^ CFUs of 10%-mannose-pretreated H10407 (CFA/I), THK38/pEU405 (CS1), DH5a/pEU588 (CS2), E116 (CS3), E106 (CS4, CS6), UM75688 (CS5, CS6), or 2423 ETP98066 (CS6) and then transferred to 95%–100% confluent monolayered Caco-2 cells (7 × 10^5^ cells; HTB-37, ATCC; Manassas, VA, USA) in a well of a 48-well Falcon tissue culture plate (Falcon; Durham, NC, USA). Similarly, 15 µL heat-inactivated serum sample pooled from the group immunized with “MecVax + CFA MEFA-II,” CFA MEFA-II, or PBS (the control group) was mixed with 3.5 × 10^5^ CFUs of ETEC bacteria JF2327 (CS7), JF3276 (CS12/CS20), JF2125 (CS14), JF2350 (CS17), or JF2101 (CS21) and then transferred to Caco-2 cells to examine antibodies derived from CFA MEFA-II against adherence from the five targeted adhesins.

After incubation in a 5% CO_2_ incubator at 37°C for 1 h, Caco-2 cells were gently washed with PBS to remove non-adherent bacteria, dislodged and lysed with 0.5% Triton (Sigma), and collected with centrifugation. The collected adherent ETEC bacteria were serially diluted, plated on LB plates, and counted for CFUs after overnight growth at 37°C. CFUs were converted to percentages by referring to the CFUs of the bacteria treated with the control mouse sera as 100%.

### Mouse serum antibody neutralization against STa and CT enterotoxicity

Sera from mice in each treatment group were examined for neutralization activities against STa and CT enterotoxicity using T-84 cells (CCL-248, ATCC) and a cyclic GMP or AMP EIA kit (Enzo Life Sciences, Farmingdale, NY, USA). As we described previously (24, 25, 28, 29), 30 µL mouse serum sample pooled from each group was mixed with 2 ng STa toxin (BEI Resources, ATCC) or 30 ng CT (Sigma; we increased CT from 10 ng to 30 ng in an attempt to differentiate neutralization activity against CT from antibodies from the vaccine antigen versus dmLT adjuvant) and incubated at room temperature for 30 min and then transferred to a well of a 24-well tissue culture plate (Falcon) containing 95%–100% confluent monolayered T-84 cells and incubated in a CO_2_ incubator for 1 h (for cGMP) or 3 h (for cAMP). Cells were rinsed thoroughly with PBS to remove extracellular cGMP or cAMP, then dislodged, and lysed with 0.5% Triton to release intracellular cGMP or cAMP. The intracellular cGMP or cAMP levels (nM, picomole per mL) were measured by following the manufacturer’s protocol (Enzo Life Science). T-84 cells in culture medium without treatment with mouse sera or toxin were also included in the assay to measure intracellular cGMP or cAMP baseline levels.

### Statistical analysis

Data of antibody titers (in log_10_) and antibody functional activities against ETEC bacterial adherence (CFUs in %) or toxin enterotoxicity (cGMP or cAMP nM) were presented in means and standard deviations and analyzed for differences between the control group and each immunization group or between the group immunized with “MecVax + CFA MEFA-II” and the group immunized with MecVax or CFA MEFA-II. One-way ANOVA (GraphPad Prism 10; San Diego, CA, USA) was used to analyze if differences between the two groups were significant, with the *P* value calculated from the *post hoc* test (Turkey’s multiple comparisons test) of less than 0.05 to indicate a significant difference.

## Data Availability

Raw data from the laboratory experiment will be made available upon request.
